# Supplementary material for: Predicting visual function by interpreting a neuronal wiring diagram
Source: Nature. 2024 Oct 2;634(8032):113–23. doi: 10.1038/s41586-024-07953-5 (PMC11446822; doi:10.1038/s41586-024-07953-5)
Supplement: Supplementary file 6 — CRF and ERF predictions for individual TmY4 and TmY9 cells. Analogous to Supplementary Data 3, but for TmY target types. Shown are the top four monosynaptic pathways, the strongest pathway passing through each of the top ten intermediary types (ranking from Extended Data Fig. 7), and the trisynaptic pathway Tm1–TmY–Dm3–TmY (see the section entitled Prediction of spatial normalization). [file 41586_2024_7953_MOESM6_ESM.zip › DataS4/TmY9qΓèÑ/720575940632352071.pdf]

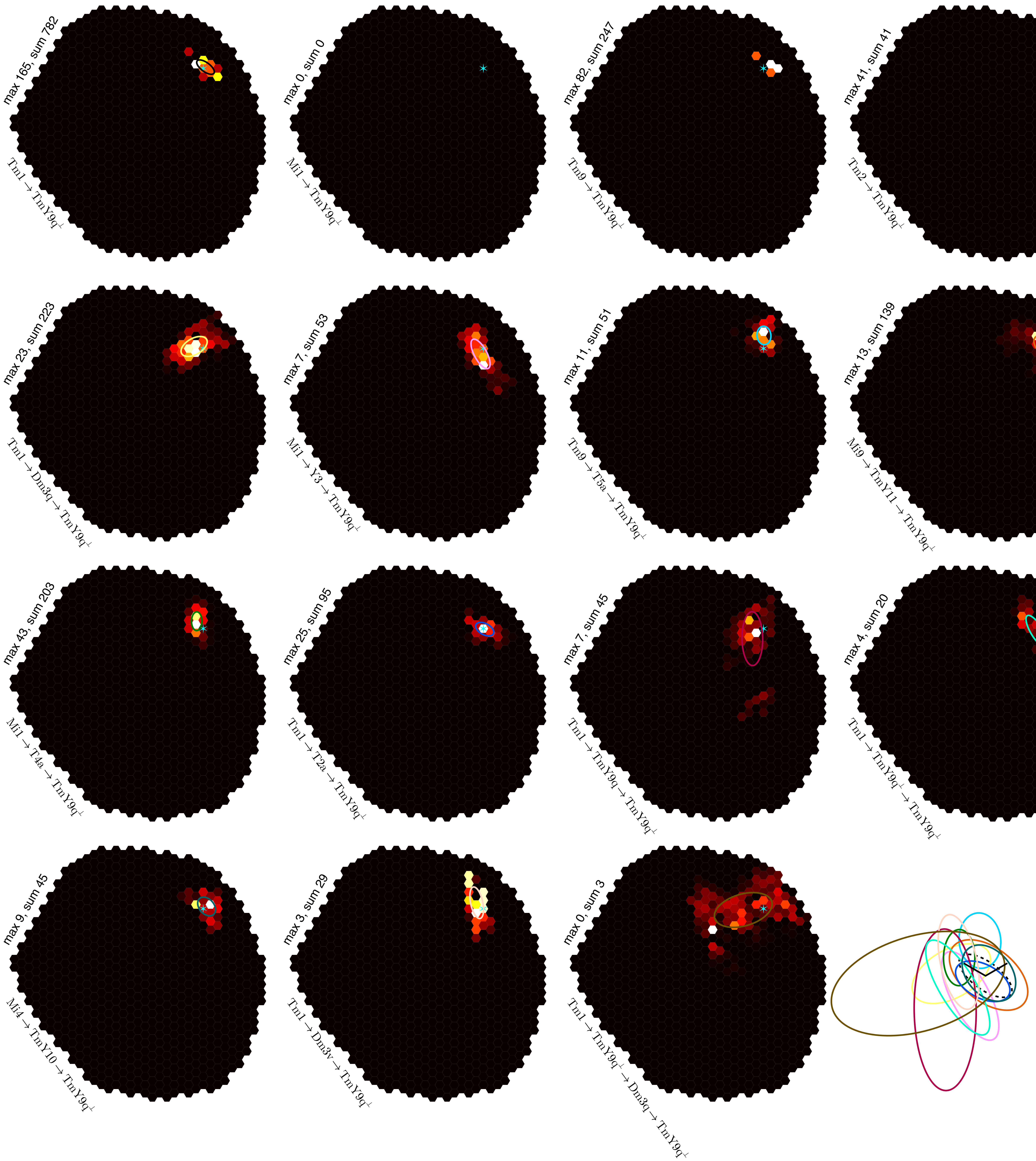
max 165, sum 782
Tm1 → TmY9q⊥
max 0, sum 0
Mi1 → TmY9q⊥
max 82, sum 247
Tm9 → TmY9q⊥
max 41, sum 41
Tm2 → TmY9q⊥
max 23, sum 223
Tm1 → Dm3q → TmY9q⊥
max 7, sum 53
Mi1 → Y3 → TmY9q⊥
max 11, sum 51
Tm9 → T5a → TmY9q⊥
max 13, sum 139
Mi9 → TmY11 → TmY9q⊥
max 43, sum 203
Mi1 → T4a → TmY9q⊥
max 25, sum 95
Tm1 → T2a → TmY9q⊥
max 7, sum 45
Tm1 → TmY9q → TmY9q⊥
max 4, sum 20
Tm1 → TmY9q⊥ → TmY9q⊥
max 9, sum 45
Mi4 → TmY10 → TmY9q⊥
max 3, sum 29
Tm1 → Dm3v → TmY9q⊥
max 0, sum 3
Tm1 → TmY9q⊥ → Dm3q → TmY9q⊥
